# Fatigue Limits Motor and Cognitive Improvements after High-intensity Exercise Prior to Balance Training over Telehealth in People with Spinocerebellar Ataxia

**DOI:** 10.63144/ijt.2025.6713

**Published:** 2025-12-12

**Authors:** Chelsea E. Macpherson, Fatima Awad, Vruta Rana, Sheng-Han Kuo, Lori Quinn

**Affiliations:** 1 Department of Biobehavioral Sciences, Teachers College, Columbia University; 2 Department of Neuroscience, Barnard College; 3 Department of Neurology, Columbia University Irving Medical Center; 4 The Initiative for Columbia Ataxia and Tremor, Columbia University; 5 Department of Rehabilitation and Regenerative Medicine (Physical Therapy), Columbia University Irving Medical Center

**Keywords:** Ataxia, Balance, Fatigue, High Intensity, Telehealth

## Abstract

**Objective:**

This pilot randomized controlled trial explored feasibility, and preliminary effects from remotely delivered high- versus low-intensity exercise prior to balance training for people with Spinocerebellar Ataxia (SCA).

**Methods:**

Twenty participants with SCA (types 1, 2, 3, or 6) were randomized to high- or low-intensity exercise (30 min), followed by balance training (30 min), delivered via telehealth twice-weekly for 8-weeks. Exercises were progressive and individualized based on ataxia severity, mobility, and home equipment. Outcomes included disease-specific measures (Scale for Assessment and Rating of Ataxia at home, Cerebellar Cognitive Affective Syndrome Scale), and fatigue (Fatigue Severity Scale) assessed at baseline, mid- and post-intervention.

**Results:**

Eighteen participants completed the intervention with high adherence. Both groups improved on disease-specific measures, with greater gains in cognition and reduced fatigue in the low-intensity group.

**Conclusions:**

Remote delivery of exercise and balance training is feasible in SCA. Fatigue may limit tolerability of higher exercise intensities. *National Institutes of Health Clinical Trials Registration Number: NCT05826171*

Spinocerebellar ataxias (SCA) are a group of rare neurogenetic disorders characterized by symptoms of ataxia that affect balance, gait, motor learning, and motor control (Diallo et al., 2020). The cerebellum is the most affected brain region, and progressive cerebellar degeneration leads to motor learning deficits that impact functional independence and capacity for neuroplastic adaptation (Mitoma et al., 2020). Strategies for clinical management focus largely on symptoms; however, they often fail to address impairments in motor learning. These motor learning impairments likely influence sustained benefits following rehabilitation interventions (Miyai et al., 2012; Morton, 2006).

Evidence supporting rehabilitation interventions for people with SCA (PwSCA) continues to evolve, with studies evaluating different exercise types (e.g., aerobic exercise, balance training, vestibular exercise, and task specific training), but also multi-modal interventions, group therapies, and alternative therapies (e.g., biofeedback, exergaming, etc.) (Bogaert et al., 2023; Heusel-Gillig & Hall, 2023; Ilg et al., 2014; Jabri et al., 2022; Marquer et al., 2014; Matsugi, 2017; Milne et al., 2017; Winser et al., 2018). Most studies have evaluated effects from individual exercises such as balance training (Keller & Bastian, 2014; Rodríguez-Díaz et al., 2018; Velázquez-Pérez et al., 2019) and more recently aerobic exercise (Barbuto et al., 2020; Di Liegro et al., 2019; Müller et al., 2020) given their potential to improve ataxia symptoms, enhance neuroplasticity, and delay neurodegeneration (Mellesmoen et al., 2018; Velázquez-Pérez et al., 2019).

Although condition specific exercise guidelines are starting to emerge (e.g., Parkinson Disease (Osborne et al., 2021), Multiple Sclerosis (Kalb et al., 2020)), many neurodegenerative conditions including cerebellar ataxias continue to follow the American College of Sports Medicine Guidelines for special populations (Riebe et al., 2018). For aerobic exercise specifically, the American College of Sports Medicine recommends engaging in moderate intensity (50–70% of heart rate maximum) exercise 30-minutes a day 3–5 days per week, for a total of 150-minutes weekly, or vigorous intensity (70–85% of heart rate maximum) exercise at least 2 days per week, for a total of 75-minutes weekly. Combinations of both moderate and high intensity exercise can also be performed to meet set guidelines (e.g., 150-minutes of moderate to vigorous physical activity (Sabag et al., 2022)). One such method is to use high-intensity interval training. High-intensity interval training includes repeated bouts of high intensity exercise (upwards of 80% of heart rate maximum) followed by varying lengths of active or passive recovery periods (Sabag et al., 2022). Results of high intensity interval training for people with cardiac conditions (e.g., coronary artery disease, post myocardial infarct, heart failure) as well as neurodegenerative conditions (e.g., Parkinson Disease, Multiple Sclerosis) have shown comparability in outcomes to continuous forms of moderate to vigorous intensity exercise the American College of Sports Medicine recommends engaging in moderate intensity (50–70% of heart rate maximum) exercise 30-minutes a day 3–5 days per week, for a total of 150-minutes weekly, or vigorous intensity (70–85% of heart rate maximum) exercise at least 2 days per week, for a total of 75-minutes weekly. Combinations of both moderate and high intensity exercise can also be performed to meet set guidelines (e.g., 150-minutes of moderate to vigorous physical activity (Sabag et al., 2022)). One such method is to use high-intensity interval training. High-intensity interval training includes repeated bouts of high intensity exercise (upwards of 80% of heart rate maximum) followed by varying lengths of active or passive recovery periods (Sabag et al., 2022). Results of high intensity interval training for people with cardiac conditions (e.g., coronary artery disease, post myocardial infarct, heart failure) as well as neurodegenerative conditions (e.g., Parkinson Disease, Multiple Sclerosis) have shown comparability in outcomes to continuous forms of moderate to vigorous intensity exercise

For balance training, interventions should incorporate components of proactive, reactive, and steady state balance for a well-rounded and multidimensional training (Gill-Body et al., 2021; Sparrow et al., 2016). Guidelines for balance training in PwSCA have not yet been established, however, gathering from other neurodegenerative conditions such as Parkinson Disease, multidimensional balance training should occur for 30–120 minutes per session, at a frequency of two to three times per week, for at least 5 to 10 weeks to see changes on condition specific motor scales, dynamic tests of balance (e.g., the Functional Gait Assessment), and measures of balance confidence (e.g., the ABC Scale) (Osborne et al., 2021). Additionally, in order to achieve the greatest functional and cognitive benefit, balance training should be guided by increasing task difficulty that reaches a person’s individual challenge point using theoretical frameworks from both Gentile’s taxonomy of tasks and the challenge point hypothesis (Bae et al., 2025; Conradsson et al., 2017; Gentile, 2000; Guadagnoli & Lee, 2004).

Studies in PwSCA and other neurological conditions, highlight the importance of moderate to vigorous intensity exercise (Ahlskog, 2011; Barbuto et al., 2020) and the importance of incorporating challenging tasks of progressive difficulty for enhanced motor learning and neuroprotective benefit (Guadagnoli & Lee, 2004; Hirsch & Farley, 2009; Hornby et al., 2020; Osborne et al., 2021). However, balance training and aerobic exercise may independently activate different neural recovery mechanisms, and combining each intervention may offer additional benefit for PwSCA. Motor priming refers to a non-conscious process in which exposure to one stimulus influences the response to a subsequent, separate stimulus, thus facilitating learning or performance (Stoykov & Madhavan, 2015). Recent literature suggests that when aerobic exercise is positioned prior to task-specific exercise (e.g., balance training) it may provide effects of “priming” that boost motor performance (Macpherson et al., 2023; Moriarty et al., 2019; Steib et al., 2018; Stoykov & Madhavan, 2015). Mechanistically this is thought to occur as aerobic exercise improves neuroplasticity, by way of increasing blood flow, oxygenation, receptor activity, and release of neurotropic factors (El-Sayes et al., 2019), while balance training increases cortical thickness and excitability (Rogge et al., 2018; Taube et al., 2007; Ueta et al., 2022). The cerebellum is heavily involved in motor learning, and degeneration of cerebellar structures disrupts neural connectivity, motor learning ability, and functional mobility (De Zeeuw & Ten Brinke, 2015). Motor priming through exercise has potential to boost motor learning and advance functional gains. To date, no rehabilitation interventions have targeted motor learning via long-term processes of motor priming with exercise in PwSCA.

This pilot randomized controlled trial was informed by prior pilot study work (Macpherson et al., 2023). The current study aimed to evaluate the feasibility and preliminary efficacy of an 8-week remote exercise intervention that compared high intensity exercise prior to balance training (HIGH-BT) to low intensity exercise prior to balance training (LOW-BT) in people with genetically confirmed SCA types 1, 2, 3, or 6.

## Methods

This pilot randomized control trial ran from April 2023 to March 2024. The study protocol was registered on the National Institute of Health Clinical Trials Registry (NCT0582617, Date of Trial Registration: 2023-03-22), and followed guidelines established by the Consolidated Standards of Reporting Trials (Boutron et al., 2017).

### Recruitment

Participants with SCA (*n*=20) were recruited from Columbia University Irving Medical Center Ataxia & Tremor Clinic, ClinicalTrials.gov, the National Ataxia Foundation (study recruitment posting, support group talks), and by word of mouth. Those who expressed interest received information by study personnel and were screened over the phone by the study principal investigator (CM).

### Eligibility Criteria

Participants were included if they were (1) age >18 years; (2) had genetic confirmation of SCA1, 2, 3, 6 or 7; (3) had a Scale for Ataxia Rating and Assessment (Schmitz-Hubsch et al., 2006) score between 8–25/40, capturing mild-moderate disease; (4) were able to walk with or without assistive devices; (5) successfully completed the Physical Activity Readiness Questionnaire (S. Thomas et al., 1992) to confirm no medical contraindications to exercise; and (6) had care partner availability during intervention. Participants were excluded if they had (1) severe non-ataxic motor symptoms measured by Inventory of Non-Ataxia Signs (Jacobi et al., 2013); (2) any visual complications associated with ataxia (e.g., retinal or optic nerve involvement); (3) spontaneous nystagmus; (4) other concurrent disease of the cerebellum (e.g., stroke, multiple sclerosis); (5) cardiopulmonary diseases that would restrict exercise; or (6) currently engaged in >3 week aerobic exercise and/or balance training. All eligible participants were enrolled in the study by the study principal investigator (CM), and signed informed consent approved by the Teachers College, Columbia University Institutional Review Board (TCIRB#23-088).

### Randomization

A block stratified randomization module was developed in REDCap (Vanderbilt University, Nashville, TN, USA) that randomized participants in blocks of four by age (> or < 65 years), and sex (male, female). Randomization was conducted using REDCap’s block randomization tool after baseline assessment. After randomization, participants were informed of their group allocation (either HIGH-BT, or LOW-BT) via secure e-mail communication (compliant with the Health Insurance Portability and Accountability Act (HIPAA)) between the study principal investigator (CM) and the participant. Participants were instructed not to disclose their group assignment in online ataxia forums or support groups to minimize contamination.

### Study Materials

Prior to baseline assessments, participants were sent a study packet containing a gait-belt to reduce fall risk, a chest-worn smart-phone holder for digital balance and functional outcome assessments via Mon4t Encephalog Balance Assessment System (Mon4t LLC, Binyamina, Israel), and a wrist-worn consumer-grade wearable device (FitBit Charge HR 5, San Francisco, CA, USA).

### Safety Considerations

All participants and their care-partners underwent a 30-minute safety training session via telehealth with the study interventionist (CM) prior to study initiation (Week 0). 1 Training included establishment of safe environments, participant guarding (Clarke et al., 2013; Kalra et al., 2004; Vloothuis et al., 2019), and medical safety (e.g., warning signs of an adverse response to exercise) (Macpherson, 2024). Additionally, participants developed an emergency action plan 2 with the interventionist, which consisted of location of exercise sessions, emergency contact name, medical providers, and local hospital of choice. These details were shared with participants’ care-partners and/or emergency contacts.

For all telehealth sessions, a HIPAA-secure automatic invite containing location, time, and type of session was sent to the participant, care-partner, or emergency contact. In the event of a medical emergency, the emergency action plan was activated. All adverse events were documented and reported to the study principal investigator, research team, and Teachers College, Columbia University IRB.

### Outcome Assessments

Primary metrics of feasibility included: (1) recruitment rate, defined as the percentage of participants referred from medical providers who then signed an informed consent and enrolled in the study; (2) retention rate, defined as the percentage of enrolled participants who completed the intervention and post-intervention assessments; (3) safety, defined as the number of adverse and serious adverse events which were reported during the trial; (4) adherence to the intervention, defined as the attendance to all sessions of the intervention; (5) cardiovascular tolerability to the intervention which was defined as meeting target heart rate reserve or rating of perceived exertion 70% of the time, and (6) acceptability, captured via a custom post-intervention questionnaire that was administered to participants via REDCap survey (see Appendix A) (Macpherson, 2024). The questionnaire consisted of 11 closed-ended questions scored on a 5-point Likert scale, that discussed four domains: overall satisfaction, interaction with the physical therapist, telehealth, and self-efficacy for exercise. There were seven open ended questions that evaluated four domains: motivation for participation, program assessment, change in function, integration of technology or telehealth.

Primary clinical outcomes were assessed by a trained clinical evaluator, who remained constant throughout the trial and was blinded to group allocation (VR). Outcomes included: (1) the Scale for Assessment and Rating of Ataxia at Home (SARAHome) (Grobe-Einsler et al., 2021) and (2) the Cerebellar Cognitive Affective Syndrome scale (CCAS Scale) (Hoche et al., 2018) both of which were video-recorded for quality assurance. The SARAHome Scale is a remote version of the original Scale for Assessment and Rating of Ataxia scale. It consists of 5 of the 8 original Scale for Assessment and Rating of Ataxia items (gait, stance, speech, nose-finger test, fast alternating hand movements). Total scores range from 0 to 28, with 0 reflecting no presence of ataxia and 28 reflecting severe ataxia. The SARAHome was found to be highly correlated with the original Scale for Assessment and Rating of Ataxia scale (*r* = 0.98, *p* < 0.0001) (Grobe-Einsler et al., 2021). The Cerebellar Cognitive Affective Syndrome scale is a 10-item scale that provides pass/fail indications per item (representing the total score out of 10 points), with a maximum raw score of 120 points. Pass/fail criteria are used to determine if a participant had possible (one failed test item), probable (two failed test items), or clinically definite (≥ three failed test items) CCAS. A cut-off score of 80/120 has been documented as criteria to obtain intervention for cognitive impairment (Hoche et al., 2018).

Secondary outcomes of functional mobility and balance included the Timed Up and Go (TUG) test (Macpherson, C.E. et al., 2025; Podsiadlo & Richardson, 1991), 30 second Sit to Stand (30 sec STS) test (Khalil et al., 2010; Polidori et al., 2024), and measures of static postural sway (neutral stance, feet together eyes open, feet together eyes closed, tandem stance, single leg stance right, single leg stance left) (Macpherson, C.E. et al., 2025). These outcomes were digitally captured using the Mon4t Encephalog Balance Assessment System (Mon4t LLC, Binyamina, Israel) (Karlinsky et al., 2022; Tchelet et al., 2019). Additionally, several patient reported outcomes were included to understand the impact of this intervention on balance confidence through the Activities Specific Balance Confidence (ABC) Scale (Powell & Myers, 1995), fatigue via the 49-item Fatigue Severity (FSS-49) Scale (Krupp et al., 1988), quality of life and perceptions of disease related function through the Neurological Quality of Life (NeuroQofL) Scale (Cella et al., 2011), Patient Reported Measure for Ataxia (PROM-Ataxia) (Schmahmann et al., 2021), and the Patient Global Impression of Change Score (PGIC) (Rampakakis et al., 2015). To understand effects of this intervention on motor adaptation, we explored the use of a remotely delivered visuomotor adaptation task (Tsay et al., 2022).

Primary outcomes of motor and cognitive function were administered over a HIPAA-secure telehealth platform (Zoom for Healthcare Inc, San Jose, CA, USA) at baseline 1 (Week 0), baseline 2 (Week 8), mid-intervention (week 12), and post-intervention (Week 16). All participants were required to have a care-partner present at baseline (Week 0), however for remaining assessments and intervention sessions the study team advised participants about the need for a care-partner based on fall risk from initial balance test scores (e.g., TUG <14 sec, stance in positions for Static Posturography >10 sec, CCAS scale raw score <80/120, and clinical judgement). All patient reported outcomes were administered via secure REDCap survey, at all four assessment points, except for the PGIC score and the post-intervention acceptability questionnaire, which were measured at post-intervention (Week 16).

### Intervention

Once randomized, all participants underwent an 8-week control period to establish a baseline and evaluate performance effects on any of the outcome measures. During this time, participants were asked to continue with their typical activities of daily living, and not to engage in any novel forms of exercise. After the control period, participants proceeded with 8-weeks of 1-on-1 remote intervention sessions delivered on Zoom Healthcare by a licensed physical therapist (CM) twice weekly, for 1 hour (16 sessions). The first half (25-minutes) of each session consisted of high intensity interval training for the HIGH-BT group, or low intensity exercise for the LOW-BT group, with a 5-minute transition period (Roig et al., 2016; R. Thomas et al., 2016), prior to the second half which consisted of 30-minutes of intensive balance training for both groups. Exercises were tailored to the participant based on ataxia severity and functional presentation (Barbuto et al., 2020), and the physical therapist (CM) provided individualized cueing to ensure safe, proper body mechanics with exercise. All sessions were recorded for intervention fidelity and adhered to guidelines for practice (Macpherson, 2024; Macpherson et al., 2023). Details of the exercise protocol for both HIGH-BT and LOW-BT groups are shown in Table 1, and case exemplars for the HIGH-BT and LOW-BT group are provided in Appendix B that detail exercise progressive exercise programs for a PwSCA who participated in this study.

Heart rate intensity for both groups was individualized, and determined by either the Karvonen Formula (Camarda et al., 2008) or the Brawner Formula (Mendes, 2021) for participants who had medications that interacted with heart rate function. Heart rate was monitored digitally during each session from Fitbit Charge HR 5 and perceived effort from subjective ratings of perceived exertion ratings on the Borg CR10 Scale. Each were obtained from participants at 3–5 minute intervals, as well as during post-intervention recovery at minutes 1, 2 and 5 (Macpherson, 2024).

Both groups underwent the same balance training program. The balance training protocol was based in part on previously used training protocols for PwSCA and other neurodegenerative conditions (Callesen et al., 2018; Gunn et al., 2015; Sparrow et al., 2016). Fitting with the Challenge Point Hypothesis, the balance training exercises focused on the skill, the learner, and the difficulty of the tasks to be learned (Guadagnoli & Lee, 2004). To optimize motor learning, balance training exercises were individualized (Barbuto et al., 2020; Statton et al., 2018), variable, progressive, and included active participation in problem solving. Guided by Gentile’s Taxonomy of Tasks (Huxham et al., 2001), vision, proprioception, and vestibular senses were integrated within a range of tasks challenging proactive, reactive and steady state balance (Gill-Body et al., 2021; Keller & Bastian, 2014; Pérez-Avila et al., 2004; Velázquez-Pérez et al., 2019).

### Statistical Analysis

Sample size was determined *a priori* from pilot data of the modified Scale for Assessment and Rating of Ataxia scale (Macpherson et al., 2023) where power was set to 0.8, *α* = 0.05 on IBM SPSS Statistics Version 29 (IBM SPSS Inc, Chicago, IL, USA), and a sample of 20 participants with SCA was estimated for this pilot randomized controlled trial study. Data analysis was performed in IBM SPSS Statistics Version 29 (IBM SPSS Inc, Chicago, IL, USA), while data visualizations were performed in R (R Core Group, Vienna, Austria) (Lin Pedersen, T, n.d.; R Core Team, (2021); Revelle, W. 2024; Singmann, H. et al., 2024; Wickham, H. et al., 2019, 2023).

Metrics of feasibility and demographic data for all participants were summarized using descriptive statistics. Primary clinical outcome measures of SARAHome, CCAS scale raw score, and PROM-Ataxia were log transformed to achieve normality. Patient reported secondary outcome measures were normally distributed, while digital recordings of functional mobility and balance were log transformed to achieve normality. Except for the PGIC score, all quantitative outcome measures were analyzed with both descriptive statistics and a repeated-measures analysis of variance using the effect of group (HIGH-BT, or LOW-BT) and time (Baseline Week 0, Baseline Week 8, Mid-Intervention Week 12). All primary and secondary outcome measures met Mauchly’s test for the assumption of sphericity (*p* > .05), except for the SARAHome, where Mauchly’s test for the assumption of sphericity was violated (χ^2^(5) = .33, *p* = .006), and therefore degrees of freedom were corrected using the Greenhouse-Geisser correction (ɛ = .65). Post-Intervention (Week 16) data were evaluated with an alpha set at *p* < .05. Post-hoc tests used Bonferroni test for the CCAS scale raw score, and the FSS-49. The PGIC score was analyzed with descriptive statistics for Week 16.

Qualitative data from the post-intervention survey was divided into analysis of Likert questions or yes/no responses, and open-ended questions. Responses to Likert questions and yes/no responses were analyzed with descriptive statistics. Responses to open ended questions were inductively coded into themes (Meissner et al., n.d.). Themes were determined by iteratively breaking down the data into smaller groups then creating codes that linked themes with interview questions. Themes were organized in a hierarchical coding frame and assigned numerical values for analysis. Any discrepancies were addressed by team discussion.

## Results

### Descriptive and Demographic Data

Participant characteristics are in Table 2. Most participants had SCA type 6 (*n*=9), followed by SCA type 3 (*n*=8), SCA type 2 (*n*=2), and SCA type 1 (*n*=1). Participants had a mean (standard deviation) disease duration of 4.87(3.29) years and mean(SD) SARAHome score of 8.71(2.68) points, suggesting motor function was in early disease stages. Participants had a mean(SD) CCAS scale raw score of 95.68(10.21), and CCAS scale total score of 8.11(1.59), suggesting probable cerebellar cognitive affective syndrome.

### Feasibility Metrics

#### Recruitment and Retention

**Figure 1:**
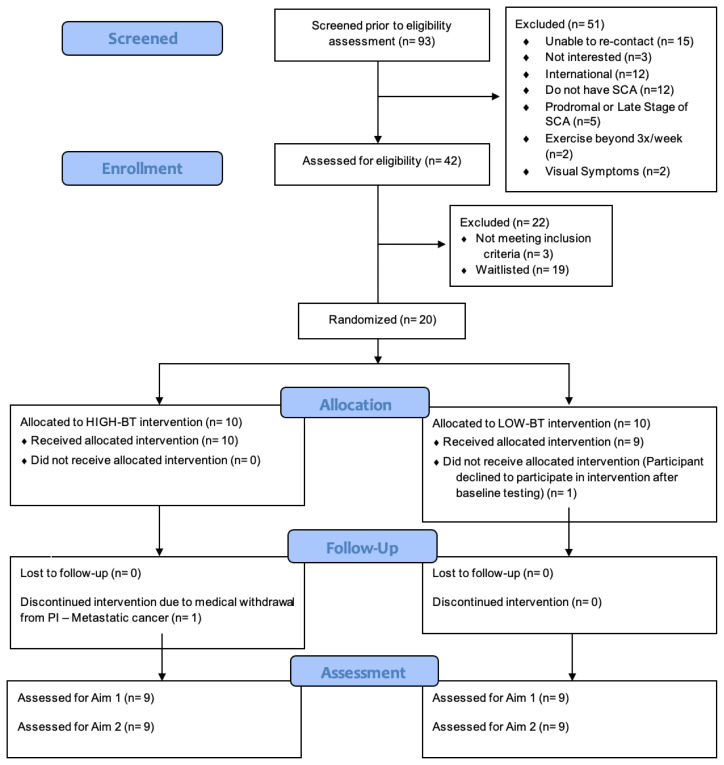
CONSORT Flow Diagram **Abbreviations:** HIGH-BT, High Intensity prior to Balance Training Group; LOW-BT, Low Intensity prior to Balance Training Group.

Participants were recruited from November 16, 2022, through September 30, 2023. Data collection was completed by March 4, 2024. Retention rate was 90% (*n*=18). Attrition rate per group was 10%, as the HIGH-BT group had one participant medically withdrawn from the study (*n*=9 for analysis), and the LOW-BT group had one participant self-withdraw (*n*=9 for analysis) (see Figure 1).

#### Acceptability

Acceptability criteria defined *a priori* was met by both groups (see Appendix 1). The post-intervention questionnaire revealed most participants preferred the option of telehealth over in-person sessions (83%, *n*=15). Analysis of Likert questions revealed intervention satisfaction was rated as mean(SD) 4.6(0.3) while therapist interaction was rated 5.0(0.0) (Likert scale 1–5; 1 = strongly disagree, 3 = neutral, and 5 = strongly agree). Participants also noted that the intervention resulted in improvements in walking or balance post-intervention (mean(SD) 4.5(0.6)), provided information regarding the management of motor symptoms (4.6(0.5)), and instilled the importance of disease-specific exercises (4.7(0.6)).

Qualitative analysis revealed that telehealth sessions were convenient, reduced time, energy, and cost involved in travelling to in-person sessions. Some participants recommended having an option for hybrid care as an idea for future trials. Primary reasons for participating in the study were to 1) maintain functional independence, 2) reduce disease severity, and 3) be proactive with research efforts. Participants expressed greatest intervention benefits from therapist interaction/support, development of a new exercise program, and the intervention building confidence for mobility. Alternatively, for aspects of the intervention that were deemed not enjoyable, participants mentioned 1) challenging motor and cognitive assessments; 2) the use of FitBit as being difficult to readily and consistently monitor heart rate throughout the intervention (e.g., difficulty navigating device prompts, delays in obtaining heart rate, etc.); and 3) the need for a longer intervention period (beyond 8 weeks) to see greater functional improvements.

#### Adverse Events

There was one adverse event of low back pain that occurred outside of the intervention (work-related) to a participant in the HIGH-BT group. The participant was cleared by a medical doctor to continue, however, the low back pain affected intervention tolerability. The intervention was modified to reduce high to moderate intensity of exercise and integrate core training as well as coaching on body mechanics for the remainder of the study (session 6/16 onward). The participant’s back pain had resolved by the end of the trial. There were no serious adverse events during the trial. This met definitions of safety defined *a priori* for this pilot randomized controlled trial.

#### Cardiovascular Tolerability

Throughout the 8-week intervention, the LOW-BT group consistently demonstrated cardiovascular tolerability, with maintenance of heart rate reserve <40%, and rating of perceived exertion between 1–3 on the Borg CR10 scale across all sessions. In contrast, the HIGH-BT group showed reduced physiological markers of cardiovascular tolerability (e.g., heart rate reserve), particularly at high over moderate intensities despite reporting rating of perceived exertion within acceptable ranges at both levels (see Table 3). This discrepancy highlights a potential disconnect between physiological and self-reported measures of exercise intensity, especially as exercise intensity increases.

#### Balance Tolerability

The HIGH-BT group consistently showed decreased tolerability to engage in balance training after aerobic exercise in comparison to the LOW-BT group. While the LOW-BT group completed mean (SD) of 10.0(2.2) balance exercises per session, the HIGH-BT group only completed 8.5(2.4) largely due to self-reported fatigue as evidenced by request of frequent rest breaks, difficulty concentrating on tasks (e.g., further explanation required, task break down). Further, LOW-BT group progressed challenge of balance exercises on 83.4(14.4) percent of sessions, while the HIGH-BT group only progressed balance exercises on 69.55(14.5) percent of sessions.

### Preliminary Efficacy of Primary Outcomes

#### SARAHome

**Figure 2:**
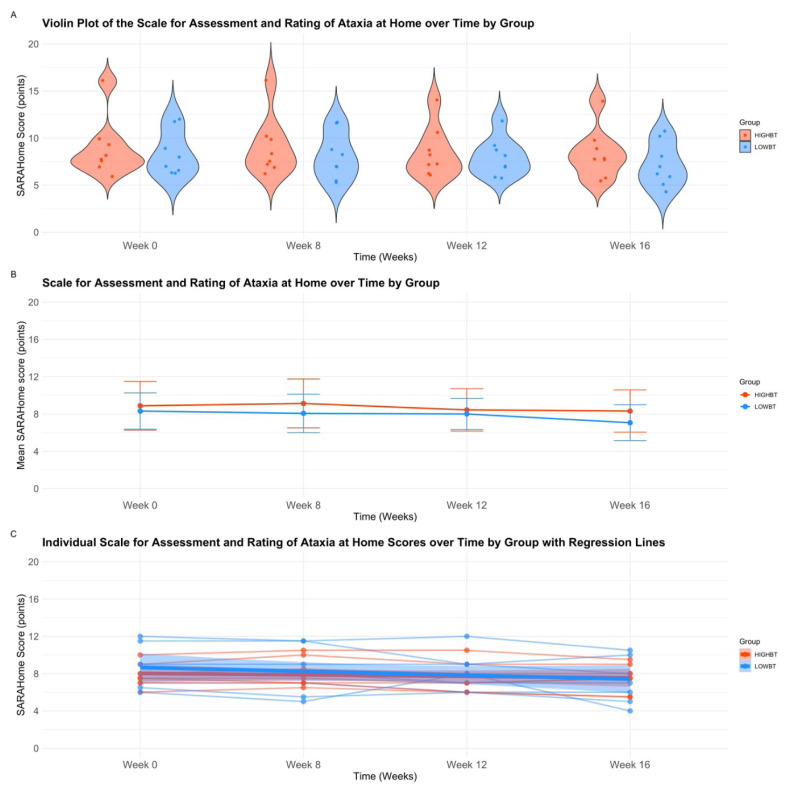
A–C Scale for Assessment and Rating of Ataxia at Home **Abbreviations:** HIGH-BT, High Intensity prior to Balance Training Group; LOW-BT, Low Intensity prior to Balance Training Group, SARAHome, Scale for Assessment and Rating of Ataxia at Home. *Note*. **A)** Violin plot with jitter overlay of Scale for Assessment and Rating of Ataxia at Home scores by group and time. This figure represents the distribution of Scale for Assessment and Rating of Ataxia at Home scores by group over baseline (Week 0), baseline (Week 8), mid-intervention (Week 12), and post-intervention (Week 16). Individual participant data is presented by points overlayed upon the violin plot. **B)** Repeated Measures analysis of variance interaction plot for Scale for Assessment and Rating of Ataxia at Home scores, evaluating group (HIGH-BT, LOW-BT), over time (Baseline Week 0, Baseline Week 8, Mid-Intervention Week 12, Post-Intervention Week 16). **C)** Scatter plot with grouped regression of individual Scale for Assessment and Rating of Ataxia at Home scores over four times points. This figure represents individual raw scores of the Scale for Assessment and Rating of Ataxia at Home across groups over baseline (Week 0), baseline (Week 8), mid-intervention (Week 12), and post-intervention (Week 16).

The SARAHome scores showed no main effect for group or time, and no interaction effects (*p*>.05) on repeated measures analysis of variance (see Table 4). Descriptive plots, and interaction plots for the SARAHome over time are displayed in Figure 2.

#### CCAS Scale Raw Score

**Figure. 3:**
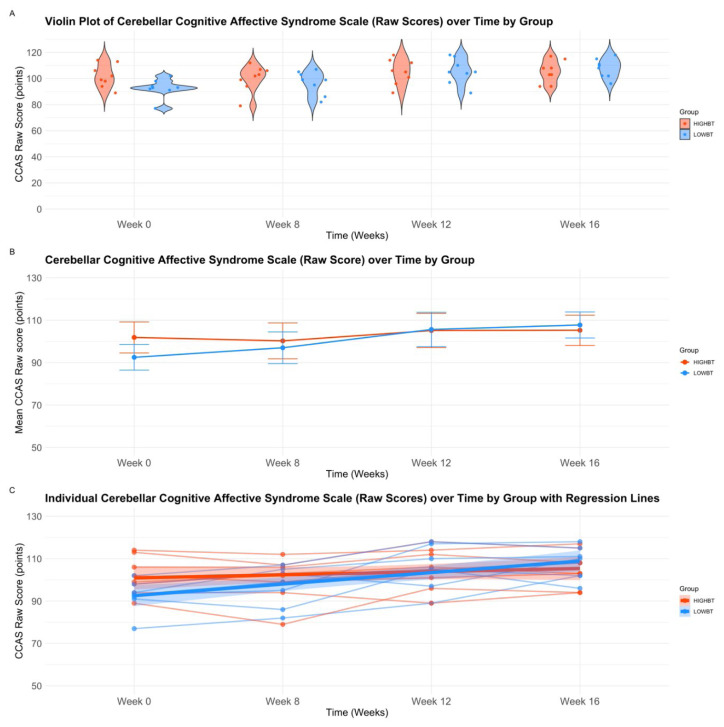
A–C Cerebellar Cognitive Affective Syndrome Scale (Raw Score) **Abbreviations:** HIGH-BT, High Intensity prior to Balance Training Group; LOW-BT, Low Intensity prior to Balance Training Group, CCAS Raw, Cerebellar Cognitive Affective Syndrome Scale – Raw Score. *Note*. **A)** Violin plot with jitter overlay of Cerebellar Cognitive Affective Syndrome scale raw scores by group and time. This figure represents the distribution of Cerebellar Cognitive Affective Syndrome scale raw scores by group over baseline (Week 0), baseline (Week 8), mid-intervention (Week 12), and post-intervention (Week 16). Individual participant data is presented by points overlayed upon the violin plot. **B)** Repeated Measures analysis of variance interaction plot for Cerebellar Cognitive Affective Syndrome scale raw scores, evaluating group (HIGH-BT, LOW-BT), over time (Baseline Week 0, Baseline Week 8, Mid-Intervention Week 12, Post-Intervention Week 16). For better visualization of effects, we have altered the scale from 0–120 (which denotes the range of possible scores on the CCAS Raw) to 50–120. **C)** Scatter plot with grouped regression of individual Cerebellar Cognitive Affective Syndrome scale raw scores over four time points. This figure represents individual raw scores of Cerebellar Cognitive Affective Syndrome scale raw scores across groups over baseline (Week 0), baseline (Week 8), mid-intervention (Week 12), and post-intervention (Week 16). For better visualization, we have altered the scale from 0–120 (which denotes the range of possible scores on the CCAS Raw) to 50–120.

There was an interaction between group and time over the 16-week intervention *F*(3, 48) = 5.16, *p* = .04. There was no effect of group *p* > .05, however there was a significant effect of time *F*(3, 48) = 19.92, *p* < .001. Post-hoc comparisons of time using a Bonferroni correction revealed significant differences between baseline Week 0 and mid-intervention Week 12 (mean difference 8.17 points, 95% CI [4.08, 12.62], *p* < .001), as well as post-intervention week 16 (mean difference 9.44 points, 95% CI [5.52, 13.37], *p* < .001). Additionally, there were significant differences between baseline Week 8 and post-intervention Week 16 (mean difference 7.28 points, 95% CI [2.40, 12.15], *p* < .01) (See Table 4, Figure 3).

#### PROM-Ataxia

**Figure 4:**
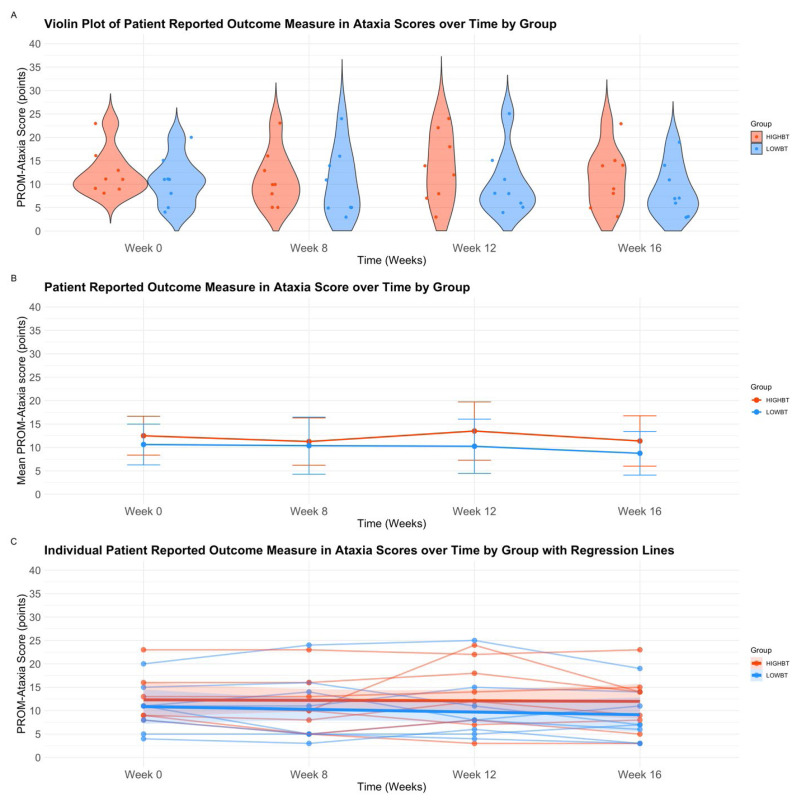
A–C Patient Reported Outcome Measure in Ataxia **Abbreviations:** HIGH-BT, High Intensity prior to Balance Training Group; LOW-BT, Low Intensity prior to Balance Training Group, PROMA, Patient Reported Outcome Measure in Ataxia. *Note*. A) Violin plot with jitter overlay of Patient Reported Outcome Measure in Ataxia scores by group and time. This figure represents the distribution of Patient Reported Outcome Measure in Ataxia scores by group over baseline (Week 0), baseline (Week 8), mid-intervention (Week 12), and post-intervention (Week 16). Individual participant data is presented by points overlayed upon the violin plot. B) Repeated Measures analysis of variance interaction plot **for** Patient Reported Outcome Measure in Ataxia scores, evaluating group (HIGH-BT, LOW-BT), over time (Baseline Week 0, Baseline Week 8, Mid-Intervention Week 12, Post-Intervention Week 16). C) Scatter plot with grouped regression of individual Patient Reported Outcome Measure in Ataxia scores over four times points. This figure represents individual raw scores of Patient Reported Outcome Measure in Ataxia scores across groups over baseline (Week 0), baseline (Week 8), mid-intervention (Week 12), and post-intervention (Week 16).

There was no interaction between group and time over the 16-week intervention (*p* > .05). There were no main effects of group or time (*p* > .05) (see Table 4). Descriptive plots, and interaction plots for the PROM-Ataxia over time are displayed in Figure 4.

### Preliminary Efficacy of Secondary Outcomes

#### Patient Reported Outcomes

**Figure. 5:**
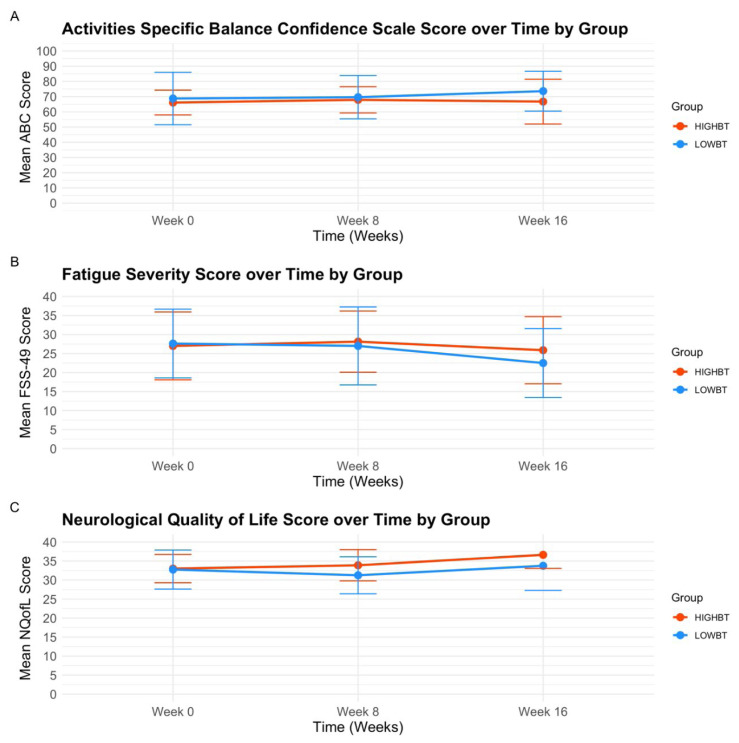
A–C Patient Reported Outcomes **Abbreviations:** HIGH-BT, High Intensity prior to Balance Training Group; LOW-BT, ABC, Activities Specific Balance Confidence Scale; FSS-49, Fatigue Severity Scale; NQofL, Neurological Quality of Life Scale. *Note*. Plot output from repeated measures analysis of variance whereby measures of Activities Specific Balance Confidence (5A), FSS-49 (5B), and NQofL (5C) are each plotted and show effects of group across three time points (Baseline Week 0, Baseline Week 8, Mid-Intervention Week 12, and Post-Intervention Week 16).

The Fatigue Severity Scale showed no main effect for group or time (*p* > .05), however there was an interaction effect between group and time, *(F* (2, 32) = 4.64, *p* = .018, partial η^2^ = .19). Post hoc pairwise comparisons used a Bonferroni correction and indicated Fatigue Severity Scale scores showed no significant differences across time points in either group (*p* > .05). There were no main effects for time or group (*p* > .05), and there were no interaction effects for the ABC scale, Fatigue Visual Analogue Scale, and NeuroQofL scale (*p* > .05), (see Table 5, Figures 5 A–C).

#### Digital Measures of Functional Mobility and Balance

For the TUG test, there was a main effect of time *F*(1, 3) = 4.36, *p* = .02, however no effect of group, and no interaction effects (*p* > .05). There were no main effects for time or group (*p* > .05), and there were no interaction effects for the 30 second sit to stand test (*p* > .05), or measures of digital posturography in any stance position (Neutral, Feet together eyes open, Feet together eyes closed, Tandem) (*p* > .05). Results for digital measures of functional mobility and balance are in Table 6, and Appendix C.

### Overall Impression of Change

The median Patient Global Impression of Change scores between the HIGH-BT and LOW-BT groups were identical with a median of 6 (IQR = [5.0, 6.0]), where a score of 6 signifies that post-intervention, participants perceived their condition to be “better” with a “definite improvement that has made a real and worthwhile difference.” There were no differences between groups (*U* = 35.5, *p* = .96, *r* = .22).

### Effect Estimates Across Outcome Measures

**Figure 6:**
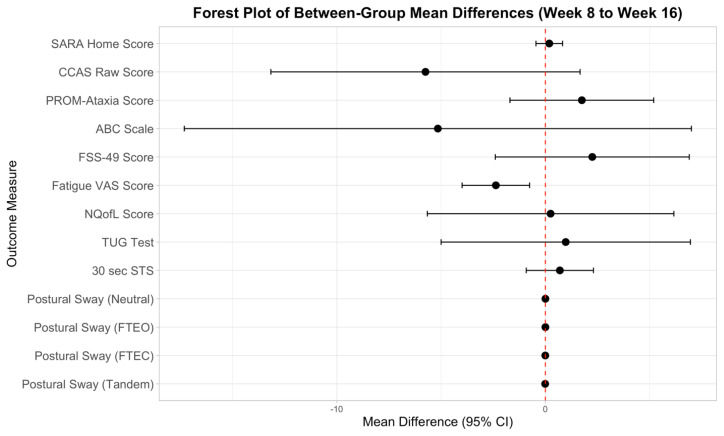
Forest Plot of Between Group Effect Estimates Across Outcome Measures **Abbreviations:** 30 sec STS, 30 second Sit to Stand Test, ABC, Activities Specific Balance Confidence Scale; CI, Confidence Interval, CCAS Raw, Cerebellar Cognitive Affective Syndrome Scale Raw Score; FSS-49, Fatigue Severity Scale 49 items; Fatigue VAS, Fatigue Visual Analogue Scale; NQofL, Neurological Quality of Life Scale; PROM-Ataxia, Patient Reported Outcome Measure in Ataxia; SARAHome, Scale for Assessment and Rating of Ataxia at Home; TUG test, Timed Up and Go test.

Figure 6 displays the mean difference between groups across outcome measures used in this pilot-Randomized Controlled Trial. Confidence intervals showed inclusion of zero across measures which suggests that the observed differences may be due to chance.

## Discussion

This pilot randomized controlled trial assessed feasibility and explored motor and cognitive outcomes after an 8-week remotely delivered intervention of high-intensity exercise prior to balance training versus low-intensity exercise prior to balance training in PwSCA. The intervention was safe and feasible, with low participant attrition, high intervention adherence and acceptance in both groups. Although our results show similar post-intervention improvements on primary endpoints and at least moderate improvements on the Patient Global Impression of Change score for both groups, the LOW-BT group showed higher tolerability for intensive balance training, greater changes in global cognitive function on the CCAS scale, and improvements in fatigue on the Fatigue Severity Scale in comparison to the HIGH-BT group at post-intervention (*p* < .01). The use of digital outcomes (e.g., digital posturography) in this study was thought to improve sensitivity of detecting subtle changes in score on standard clinical measures (e.g., SARAHome). However, no observable differences were found across groups. The use of digital assessments in PwSCA may not be sensitive to change during an 8-week exercise trial, and instead, may be better served to detect changes longitudinally. Overall, these findings reveal an emerging story of fatigue, exercise intensity and tolerability with unique challenges to capture subtle changes in function after this remotely delivered exercise trial.

Telehealth provides access to medical services within a person’s home environment, therefore reducing common barriers seen within a clinical environment (e.g., mobility impairments, time, transportation, financial burdens, care-partner availability, etc.) (Barbuto et al., 2020; Ben-Pazi et al., 2018; Chirra et al., 2019; De Marchi et al., 2021). However, telehealth has its own barriers for use, and so, considerations should be made for access to technology, technological literacy, impact of cognition, functional independence and fall risk, environmental constraints, validity and reliability of remotely delivered outcomes, as well as the availability of a care-partner for assessment and intervention delivery (Ben-Pazi et al., 2018; Chirra et al., 2019; Macpherson, 2024). In this study, we employed care-partner training, an emergency protocol, cut-offs for cognitive function, and fall risk pertaining to the involvement of a care-partner during assessments and/or intervention sessions. For safe assessment and intervention delivery, supervision was provided by a care-partner (if a participant was deemed fall risk), and a licensed physical therapist delivered the intervention. From our efforts to promote safety, there were no adverse events for assessment delivery, and one adverse event that occurred outside the intervention session (e.g., recurrence of low back pain for a participant while at work) that resolved by incorporating body mechanics and core stability to remaining sessions.

Despite high levels of intervention acceptance from both groups, participants in the HIGH-BT group showed less intervention tolerability than their intervention counterparts (LOW-BT). For aerobic exercise, as intensity rose, the HIGH-BT group showed greater difficulty maintaining target heart rate and rating of perceived exertion. For balance training, although both groups were matched for time and task-intensity, group differences revealed the HIGH-BT group showed an appreciable decrease in their capacity to engage in balance training after aerobic exercise. Specifically, participants in the HIGH-BT group requested more frequent breaks, had difficulty concentrating on complex balance tasks (e.g., required task breakdown, additional explanations) and completed less balance exercises per session than those in the LOW-BT group. Exercise tolerability may be limited at high intensities for PwSCA, and these higher intensities may impair further engagement in intensive forms of multi-modal exercise. Although higher intensity aerobic exercise is often recommended in people with neurodegenerative conditions (e.g., vigorous exercise at >75% HR max), current findings suggest that PwSCA may face challenges sustaining these workloads. These observations highlight the importance of prescribing exercise intensity to match individual capabilities, particularly when programs require sustained physiological effort.

Fatigue is a common and disabling symptom for PwSCA (Brusse et al., 2011; Lai et al., 2024). Previous studies have reported that PwSCA who reported fatigue on the Fatigue Severity Scale had increased ataxia severity, mood disorders (e.g., depression), and decreased quality of life throughout their disease course. (Lai et al., 2024) Although awareness for fatigue in PwSCA is gaining traction, research into its underlying neural mechanisms remains in stages of early development (Casamento-Moran et al., 2023). Emerging literature suggests that decreased cerebellar excitability leads to decreased perceptions of physical fatigue and worsened motor control (Casamento-Moran et al., 2023). which impact initial metabolic responses to acute forms of exercise in PwSCA (Barbuto et al., 2020; Lai et al., 2024; Macpherson, 2024). These factors were accounted for in this pilot randomized controlled trial with a ramped exercise protocol that used individualized target heart rate zones and ratings of perceived exertion. Balance training programs were also tailored to the individual based on ataxia severity and functional impairments (Barbuto et al., 2020; Lai et al., 2024). However, despite these efforts our findings suggested that although exercise may play a role in mitigating fatigue at lower intensities, decreased perceptions of physical fatigue may have contributed to over-work at higher intensities of and served as a rate limiting factor for exercise tolerability. As proposed in work by Casamento-Moran et al., (2023), fatigue and performance-related processes may compete for the same cerebellar resources, suggesting a need to better integrate fatigue awareness and mitigation strategies when engaging in higher intensities of exercise, or with intensive multimodal training programs (Casamento-Moran et al., 2023)

In the past decade, greater emphasis has been placed upon increasing intensity across exercise type to promote neuroplasticity (Hortobágyi et al., 2021; Miyai, 2012; Rogge et al., 2018; Sparrow et al., 2016). However, a growing body of evidence is showing that perhaps the highest intensities of exercise are not necessary to induce the greatest gains in function and neuroplasticity (Hugues et al., 2022; Macpherson, 2024; Statton et al., 2015; Steib et al., 2018; Wanner et al., 2020; Youssef et al., 2024; Zoladz et al., 2014). The premise of the current study stemmed from motor priming literature, which often uses high intensity aerobic exercise prior to intensive task specific training (e.g., balance training) to elicit changes in motor learning and behavior (T. A. Moriarty et al., 2019; Sivaramakrishnan et al., 2022; Stoykov & Madhavan, 2015). However, individual differences, disease severity, decreased physical capacity, and presence of fatigue are important considerations for clinicians and researchers as they may not only contribute to the development of secondary comorbidities, but also an inability to safely tolerate higher forms of exercise intensities. Emerging literature is revealing that effects of motor priming can not only be achieved with moderate intensities of aerobic exercise (T. A. Moriarty et al., 2019) in people with Parkinson disease (Steib et al., 2018) and adult control populations (T. Moriarty et al., 2022), but that moderate and high intensities may play different roles for skill acquisition and retention (T. Moriarty et al., 2022; Statton et al., 2015; Swarbrick et al., 2020). As PwSCA in our study had greater tolerability of moderate intensity aerobic exercise compared to high intensity aerobic exercise, future work should explore moderate intensity aerobic exercise prior to balance training as a form of motor priming.

## Limitations

Our sample included adults with early to middle stages of ataxia severity (Scale for Assessment and Rating of Ataxia scale 8–25/40), with known impairments in gait, balance, and functional mobility. Digital outcomes may be better served among those in prodromal or early disease states (Scale for Assessment and Rating of Ataxia scale <8/40), while changes in later stages may be better assessed with use of clinical measures such as the Scale for Assessment and Rating of Ataxia. In addition, the sample size for this pilot randomized controlled trial was underpowered to determine preliminary intervention efficacy. Although sample size estimates were informed *a priori* from our pilot trial (Macpherson, 2024), our post-hoc sensitivity analysis revealed that our results were significantly smaller than expected, and that we did not have at least 80% power to detect effects without Type II error.

## Conclusion

This study supports the feasibility of a telehealth-delivered exercise intervention for PwSCA. Low intensity exercise prior to progressive balance training was feasible and may improve disease-specific outcomes and fatigue in PwSCA, while high intensity exercise appeared to influence fatigue, thus limiting intervention tolerability. Future research should explore the use of low versus moderate intensity exercise prior to intensive balance training and aim to optimize exercise prescription and mitigate fatigue in PwSCA.

## Figures and Tables

**Table 1:** Overview of Intervention Protocol

Frequency Intensity Type and Time Principle	HIGH-BT Group: High Intensity Interval Training Protocol	LOW-BT Group: Low Intensity Exercise Protocol	HIGH-BT and LOW-BT groups: Balance Training Protocol
**Frequency**	------------------------2x/week for 8-weeks -----------------------		
**Intensity**	*Ramped Protocol High intensity: 65–85% heart rate reserve; rating of perceived exertion 7–9 Moderate intensity: 50–64% heart rate reserve; rating of perceived exertion 3–6	Low intensity: <40% heart rate reserve; rating of perceived exertion <3	Using the Challenge Point Hypothesis participants were prescribed exercises with progressive high intensity challenge to form failure, or reported fatigue
**Time**	Total Time: 25-min Warm-up: 5-minutes high intensity interval training protocol: 15 minutes - 5 × 3-minute intervals - 3-minute interval = 1-minute high intensity + 2 minutes moderate intensity Cool-down: 5 minutes *5-minute transition prior to Balance Training	Total Time: 25-min Warm-up: 5-minutes Low-intensity exercise protocol: 15-minutes continuous exercise Cool-down: 5-minutes *5-minute transition prior to Balance Training	Total Time: 30-minutes
**Type**	The type of exercise will vary based on equipment availability, ataxia severity and functional status.		
	Examples of high intensity exercises include: Walking (i.e., on flat or hilly surfaces, or treadmill) Recumbent cycle ergometer Elliptical Bodyweight exercises (i.e., step-ups and squats to time) Resistance training exercises (i.e., dumbbell renegade rows, floor to stand)	Examples of low intensity exercises include: Core Strength (e.g., Deep core activation) Yoga (e.g., Hatha Yoga) Stretching (e.g., postural restoration) Therapeutic Exercises (e.g., seated total body resistance band exercises, calisthenics)	Examples of balance exercises include: Static Standing (e.g., semi-tandem, tandem stance) Dynamic Standing (e.g., sit to stand, reaching with hand or foot outside base of support) Walking Balance (e.g., forward to backward walking, side stepping, walking with head turns) Sensory Integration (e.g., visual, proprioception, vestibular integration) All exercise domains were progressed according to manipulation of physical support (upper extremity assist), base of support (wide stance, neutral stance, etc.), visual input (sunglasses, eyes closed), proprioceptive input (stance on firm or pliable surface), vestibular input (head turns)
**Total** **Weekly Volume**	60-minute sessions × 2 days per week = 180-minutes/week		

Abbreviations: HIGH-BT, High-intensity exercise prior to balance training group; LOW-BT, Low intensity exercise prior to balance training group.

**Table 2:** Participant Characteristics

Outcome	Total (N=20)	High intensity exercise prior to balance training group (*n*=10)	Low intensity exercise prior to balance training group (*n*=10)
Age (years)	58.1 (13.5)	57.4 (14.7)	59.7 (12.5)
Sex (freq)			
Male	6	4	2
Female	14	6	8
Weight (kg)	72.92 (13.8)	72.9 (14.5)	72.6 (13.1)
Height (cm)	165.0 (14.2)	164.2 (17.3)	166.8 (10.6)
Race/Ethnicity (freq)			
White	13	4	9
Black	4	3	1
Asian	3	3	0
Education (freq)			
High School	1	1	0
Associates	1	0	1
Bachelors	11	5	6
Masters	6	4	2
Doctorate	1	0	1
Type of SCA (freq)			
SCA Type 1	1	0	1
SCA Type 2	2	1	1
SCA Type 3	8	5	3
SCA Type 6	9	4	5
SCA Type 7	0	0	0
Disease Duration (years)	4.87(3.29)	4.41(4.07)	5.34(2.39)
SARAHome /28 (points)	8.71 (2.68)	9.20 (3.07)	8.17 (2.22)
CCAS scale raw score /120 (points)	95.68 (10.21)	97.6 (12.27)	93.56 (7.47)
CCAS scale total score /10	8.11 (1.59)	8.10 (7.47)	8.11 (0.93)
Falls/year (freq)			
None	4	2	2
Once in a year	3	2	1
Twice or more	12	5	7
History of Rehabilitation Services (freq)			
Yes	10	6	4
No	10	4	6

**Abbreviations:** number of participants (N/n), Cerebellar Cognitive Affective Syndrome Scale (CCAS), Scale for Assessment and Rating of Ataxia at Home (SARAHome), Spinocerebellar Ataxia (SCA).

**Table 3:** Intervention Tolerability as Measured by Heart Rate and Rating of Perceived Exertion, Presented as Mean(SD)

Group	Level of Intensity	Percent Time at target heart rate reserve Intensity	Percent Time at target rating of perceived exertion
LOW-BT (*n*=9)	Low Intensity *(Target: heart rate reserve <40%, rating of perceived exertion 1–3)*	100.0(0.0)	96.3(5.8)

HIGH-BT (*n*=9)	Moderate Intensity *(Target: heart rate reserve 50–64%, rating of perceived exertion 4–5)*	64.4(32.3)	92.7(14.6)
	High Intensity *(Target: heart rate reserve 65–85%, rating of perceived exertion 6–7)*	53.4(33.9)	86.4(19.2)

*Note*. Heart rate was collected using the Fitbit Charge HR 5, and Heart Rate Reserve was calculated per participant using either the Karvonen or Brawner formulas. Rating of Perceived Exertion was assessed using the modified Borg Category-Ratio-10 scale. For analysis, heart rate was averaged over defined intervals: 5-minute segments in the LOW-BT group, and alternating 2-minute (moderate intensity), and 1-minute (high intensity) in the HIGH-BT group totaling 15-minutes of work per session. rating of perceived exertion was recorded at the end of each interval. Warm-up and cool-down periods (5-minutes each) were excluded. heart rate reserve and rating of perceived exertion were compared to *a priori* targets and coded as meeting or not meeting the target (yes/no). These were averaged per session, per participant, and across the intervention to obtain percentage of time spent at target heart rate reserve and rating of perceived exertion by group.

**Table 4:** Descriptive Statistics Across Baseline Week 0, Baseline Week 8, and Post-Intervention for Disease-Specific Measures

Patient Reported Outcome Measure		High intensity exercise prior to balance training					Low intensity exercise prior to balance training				Group Comparison		
		Baseline Week 0	Baseline Week 8	Mid-Intervention Week 12	Post-Intervention Week 16		Baseline Week 0	Baseline Week 8	Mid-Intervention Week 12	Post-Intervention Week 16	Mean Difference	Confidence Interval	Sig.
	*n*	Mean (SD)	Mean (SD)	Mean (SD)	Mean (SD)	*n*	Mean (SD)	Mean (SD)	Mean (SD)	Mean (SD)		[CI]	*p*
SARA Home (/28 points)	9	8.78 (2.94)	9.00 (2.96)	8.17 (2.67)	8.17 (2.57)	9	8.17 (2.22)	8.06 (2.51)	8.22 (1.86)	7.22 (2.21)	−.20	[−.73, .39]	.54
CCAS scale raw score (/120 points)	9	100.22 (9.59)	99.44 (9.79)	104.00 (9.64)	104.33 (8.46)	9	93.56 (7.47)	98.67 (9.73)	106.11 (9.17)	108.33 (7.09)	4.78	[−2.09, 11.60]	.17
PROM-Ataxia (/40 points)	9	12.22 (4.71)	11.11 (5.67)	13.00 (7.12)	11.00 (6.12)	9	12.44 (7.32)	12.22 (8.78)	12.00 (8.34)	10.67 (7.76)	−1.44	[−4.50, 1.61]	.33

* Denote statistical significance.

*Note*. Higher scores on the Cerebellar Cognitive Affective Syndrome (CCAS) scale raw score indicate greater cognitive function. Higher scores on the Scale for Assessment and Rating of Ataxia at Home scale (SARAHome) indicate greater disease severity. Lower scores on the Patient Reported Outcome Measure in Ataxia (PROM-Ataxia) indicate less disease impact.

**Table 5:** Descriptive Statistics Across Baseline Week 0, Baseline Week 8, and Post-Intervention for Patient Reported Outcome Measures

Patient Reported Outcome Measure		High intensity exercise prior to balance training					Low intensity exercise prior to balance training				Pairwise Comparison **		
		Baseline Week 0	Baseline Week 8	Mid-Intervention Week 12	Post-Intervention Week 16		Baseline Week 0	Baseline Week 8	Mid-Intervention Week 12	Post-Intervention Week 16	Mean Difference Week 8–16	Confidence Interval	Sig. (*p*)
	*n*	Mean (SD)	Mean (SD)	Mean (SD)	Mean (SD)	*n*	Mean (SD)	Mean (SD)	Mean (SD)	Mean (SD)		[CI]	
ABC(/100%)	9	63.26 (12.47)	64.86 (13.29)	69.61 (9.78)	65.83 (16.68)	9	64.23 (23.55)	65.00 (21.08)	65.21 (19.37)	68.47 (22.52)	1.94	[−9.81, 13.70]	.73
FSS-49 (/64 points)	9	26.33 (10.16)	27.56 (9.15)	29.78 (8.54)	26.22 (9.94)	9	28.22 (10.28)	27.78 (11.70)	24.78 (10.75)	23.67 (10.74)	−2,78	[−7.35, 1.79]	.22
Fatigue Visual Analogue Scale (/10 points)	9	6.11 (2.67)	5.67 (2.92)	5.67 (2.55)	5.78 (3.14)	9	5.56 (2.60)	5.33 (2.87)	6.56 (2.55)	6.89 (2.85)	1.44	[−.81, 3.69]	.20
Neuro QofL Scale (/40 points)	9	33.00 (4.15)	33.00 (5.29)	33.89 (5.82)	35.33 (5.57)	9	31.9 (6.29)	30.00 (6.61)	30.11 (6.97)	32.56 (8.08)	.22	[−5.01, 5.45]	.93

* Denote statistical significance.

** Based on Estimated Marginal Mean

*Note*. Higher scores on the Activities Specific Balance Confidence (ABC) scale indicate higher balance confidence. Lower scores on the Fatigue Severity Scale (FSS-49) indicate less fatigue. Higher scores on the Fatigue Visual Analogue Scale indicate less fatigue. Higher scores on the Neurological Quality of Life (NeuroQofL) Scale indicate higher quality of life.

**Table 6:** Descriptive Statistics Across Baseline Week 0, Baseline Week 8, and Post-Intervention for Digitally Recorded Functional Mobility and Balance Outcome Measures

Digitally Reported Outcome Measure		High intensity exercise prior to balance training					Low intensity exercise prior to balance training				Pairwise Comparisons **		
		Baseline Week 0	Baseline Week 8	Mid-Intervention Week 12	Post-Intervention Week 16		Baseline Week 0	Baseline Week 8	Mid-Intervention Week 12	Post-Intervention Week 16	Mean Difference Week 8–16	Confidence Interval	Sig.
	*n*	Mean (SD)	Mean (SD)	Mean (SD)	Mean (SD)	*n*	Mean (SD)	Mean (SD)	Mean (SD)	Mean (SD)		[CI]	(p)
TUG Test (sec)	9	20.82 (7.11)	23.29 (9.22)	24.57 (11.06)	24.35 (9.48)	9	19.91 (10.27)	20.29 (9.90)	18.07 (8.82)	19.67 (9.59)	−1.68	[−7.07, 3.71]	.52
30 sec STS Test (reps)	9	9.11 (1.54)	8.22 (2.17)	7.89 (3.59)	9.22 (0.97)	9	9.00 (2.88)	9.13 (2.60)	8.00 (4.72)	9.38 (2.77)	−.75	[−2.22, .72]	.30
Digital Postural Sway Neutral Stance (m/s^3^)	9	4.19×10^−3^ (1.7×10^−3^)	4.35×10^−3^ (1.44×10^−3^)	4.13×10^−3^ (9.96×10^−4^)	4.31×10^−3^ (9.39×10^−4^)	9	3.82×10^−3^ (9.34×10^−4^)	5.05×10^−3^ (3.87×10^−3^)	3.89×10^−3^ (9.20×10^−4^)	3.87×10^−3^ (8.92×10^−4^)	−1.28×10^−3^	[−.004, .002]	.40
Digital Postural Sway Feet Together Eyes Open Stance (m/s^3^)	9	6.10×10^−3^ (1.7×10^−3^)	7.90×10^−3^ (3.10×10^−3^)	7.29×10^−3^ (3.00×10^−3^)	6.58×10^−3^ (2.45×10^−4^)	9	1.05×10^−2^ (8.40×10^−3^)	7.68×10^−3^ (5.85×10^−3^)	8.10×10^−3^ (6.07×10^−3^)	5.55×10^−3^ (1.98×10^−4^)	−8.0×10^−4^	[−.005, .004]	.72
Digital Postural Sway Feet Together Eyes Closed Stance (m/s^3^)	9	1.34×10^−2^ (6.78×10^−3^)	1.68×10^−2^ (9.32×10^−3^)	1.46×10^−2^ (6.65×10^−3^)	1.23×10^−2^ (4.49×10^−3^)	9	1.59×10^−2^ (1.03×10^−2^)	1.50×10^−2^ (1.36×10^−2^)	1.92×10^−2^ (1.60×10^−2^)	1.58×10^−2^ (1.41×10^−2^)	−5.0×10^−3^	[−.006, .009]	.04*
Digital Postural Sway Tandem Stance (m/s^3^)	9	2.14×10^−2^ (9.29×10^−3^)	2.14×10^−2^ (1.12×10^−2^)	1.64×10^−2^ (6.39×10^−2^)	1.52×10^−2^ (7.94×10^−3^)	9	2.55×10^−2^ (1.93×10^−2^)	2.23×10^−2^ (1.11×10^−2^)	2.22×10^−2^ (1.57×10^−2^)	2.82×10^−2^ (1.79×10^−2^)	1.10×10^−2^	[−.008, .024]	.07

* Denote statistical significance.

** Based on Estimated Marginal Mean

*Note*. Lower scores on the Timed Up and Go (TUG) test indicate better functional mobility and dynamic balance. Higher repetitions on the 30 sec Sit to Stand test (30 sec STS) indicate better functional mobility and muscular endurance. Smaller values of accelerometry (m/s3) indicate less postural sway.

## Data Availability

The data are available on request from the lead author.

## Footnotes

1 Safety Training presentation is available upon request of the lead author.

2 The Emergency Action Plan is available upon request of the lead author.

## Appendix A

**Table 1:** Likert Questions from Post-Intervention Acceptability Questionnaire

Likert Questions Regarding Intervention	Total Sample Ranking (N=18)	High Intensity Exercise Prior to Balance Training Group Ranking (*n*=9)	Low Intensity Exercise Prior to Balance Training Group Tanking (*n*=9)
1. I felt adequately supported from the therapist throughout the exercise program.	4.94(0.24)	5.00(0.00)	4.88(0.35)
2. I felt more motivated to exercise after completing the program.	4.65(0.60)	4.78(0.44)	4.50(0.76)
3. I knew I could ask the therapist if I had questions.	5.00(0.00)	5.00(0.00)	5.00(0.00)
4. The intervention helped me understand the importance of exercise for cerebellar ataxia.	4.71(0.59)	4.67(0.50)	4.75(0.71)
5. The therapist was responsive to my questions and any concerns.	5.00(0.00)	5.00(0.00)	5.00(0.00)
6. This intervention helped me to improve my exercise patterns.	4.59(0.62)	4.67(0.71)	4.50(0.53)
7. This intervention helped me managed my (motor) symptoms in relation to my cerebellar ataxia.	4.56(0.51)	4.67(0.50)	4.43(0.53)
8. I felt a change in my level of physical functioning and independence after this intervention.	4.38(0.88)	4.38(0.92)	4.38(0.92)
10. I noticed changes with respect to my walking, or balance, after engaging in this intervention.	4.47(0.62)	4.67(0.50)	4.25(0.71)
11. I am confident I can track my exercise progress.	4.24(0.66)	4.22(0.67)	4.25(0.71)
12. I intend to continue exercising regularly for the next 2 months.	4.81(0.40)	4.89(0.33)	4.71(0.49)
Average Satisfaction with Intervention	4.62(0.32)	4.68(0.33)	4.53(0.32)

*Note*. Results from Post-Intervention Questionnaire. Each item is scores on Likert Scale ranging from 1–5 where a score of 1 represents Strong Disagreement, 3 is neutral, and 5 is Strong Agreement. Findings are presented as mean(SD).

## Appendix B

**Case Example: Mid-Stage Cerebellar Ataxia randomized to the High Intensity prior to Balance Training Group**

The table below represents a brief case study on a participant enrolled in the PRIME-Ataxia pilot Randomized Controlled Trial who has mid-stage ataxia. This person was randomized to the high intensity exercise prior to balance training group for the intervention phase. Below, we outline the intervention from baseline through to their final session with progressive exercises. We also describe the materials this person used in their home setting, and how we adapted the intervention over telehealth specifically to their needs. For this intervention, we took into consideration the person’s disease stage, functional mobility and balance scores, level of fitness, as well as considerations to the environment in which they would be exercising (e.g., safety, materials, presence of a care-partner consistently, etc.). At the end of the table, we briefly discuss their individual outcomes, as well as their perspectives on engaging within this type of study.

**Table:**

**PRIME-Ataxia Pilot Randomized Controlled Trial: Mid-Stage Case 1**		
Group	High Intensity exercise prior to Balance Training (HIGH-BT)	
Case Detail	P1: Pt is a 74-year old White female, with genetic confirmation of Spinocerebellar Ataxia (SCA) type 6 diagnosed 10 years prior to this study. Her symptoms began approximately 19 years prior, with mild dizziness and imbalance. She reports no prior family history of SCA type 6 that she is aware of. P1 is a retired healthcare administrator, who resides with her husband (care-partner). She engages light physical activity by taking her dog for a walk each day and goes to physical therapy 1–2x/week. At baseline she did not have a formal exercise routine, and she was not otherwise physically active outside activities of daily living. For baseline mobility, P1 ambulated in the community and at home with a rollator and service dog. In tight spaces, she would resort to furniture grabbing. Prior to the study, P1 had two falls within the past year. No injuries were sustained with these falls, however both occurred in low light environments. P1’s husband was on-site and available as needed for assessments and the intervention.	
Aerobic Exercise	**Baseline Plan**	**Plan Progressed**
	*All participants underwent a ramped protocol whereby they started both moderate and high intensities of exercise at 10%, then 5% less than target heart rate for the first 3 weeks, of the intervention as tolerable*. Karvonen Formula was used to predict target heart rate reserve per session, and Borg Rating of Perceived Exertion Scale was used in conjunction to estimate effort. **Resting heart rate:** 74 bpm **Target Moderate heart rate reserve (50–60%):** 110–117 bpm **Target Moderate rating of perceived exertion:** 4–5 **High (60–85%heart rate reserve):** 117–132 bpm **Target High rating of perceived exertion:** 6–7 **Warm up (5 min):** Upright Exercise Bike, Resistance 5, Revolutions per minute 60–80, Continuous Peddling, heart rate <110 bpm. **Moderate Intensity 1 (2 min)** Upright Exercise Bike, Resistance 5–7, Revolutions per minute 80–100, Continuous Peddling. **High Intensity 1 (1 min)** Upright Exercise Bike, Resistance 8, Revolutions per minute 100, Continuous Peddling. **Moderate Intensity 2 (2 min)** Upright Exercise Bike, Resistance 5–7, Revolutions per minute 80–100, Continuous Peddling. **High Intensity 2 (1 min)** Upright Exercise Bike, Resistance 8, Revolutions per minute 100, Continuous Peddling. **Moderate Intensity 3 (2 min)** Bimanual grip, overhead tricep press with 10 lb dumbbell ×30 sec. Bimanual grip forward flexion with 5 lbs dumbbell ×30 sec. Unilateral scaption to shoulder height with 3 lbs dumbbell ×30 sec bilaterally (*cue for muscle control). **High Intensity 3 (1 min)** Sit to stand ×1min (*cue, how many can you complete in 60 seconds). **Moderate Intensity 4 (2 min)** Standing with wide base of support, latissimus dorsi pull down with green TheraBand^TM^ ×1 minute. Standing with wide base of support rows with green TheraBand^TM^ ×1 minute. **High Intensity 4 (1 min)** Seated diagonal cross body chops with 3 lbs dumbbell ×30 sec each side. **Moderate Intensity 5 (2 min)** Side stepping with green TheraBand^TM^ around knees (holding on to wall) ×1 min. Pulsing mini lunges ×30 sec per side (holding on to chair). **High Intensity 5 (1 min)** Deadlifts with 10lbs dumbbell (knees backed to a chair) ×1 min. **Cool Down (5 min):** Diaphragmatic breathing ×5 repetitions. Diaphragmatic breathing with Transverse abdominus contraction ×10 repetitions. Diaphragmatic breathing with Transverse abdominus and pelvic floor contraction ×10 repetitions. Transverse Abdominus contraction with Marching ×10 repetitions. Pelvic Floor elevators ×5 repetitions.	Karvonen Formula was used to predict target heart rate per session, and Borg Rating of Perceived Exertion Scale was used to target rating of perceived exertion per session. **Resting heart rate:** 73 bpm **Target Moderate heart rate reserve (50–60%):** 111–120 bpm **Target Moderate rating of perceived exertion:** 4–5 **High heart rate reserve (60–85%):** 121–136 bpm **Target High rating of perceived exertion:** 6–7 **Warm up (5 min):** Upright Exercise Bike, Resistance 5, Revolutions per minute 60–80, Continuous Peddling, heart rate <110 bpm. **Moderate Intensity 1 (2 min)** Upright Exercise Bike, Resistance 7–8, Revolutions per minute 80–100, Continuous Peddling. **High Intensity 1 (1 min)** Upright Exercise Bike, Resistance 9–10, Revolutions per minute 100, Continuous Peddling. **Moderate Intensity 2 (2 min)** Upright Exercise Bike, Resistance 7–8, Revolutions per minute 80–100, Continuous Peddling. **High Intensity 2 (1 min)** Upright Exercise Bike, Resistance 9–10, Revolutions per minute 100, Continuous Peddling. **Moderate Intensity 3 (2 min)** Upright Exercise Bike, Resistance 7–8, Revolutions per minute 80–100, Continuous Peddling. **High Intensity 3 (1 min)** Upright Exercise Bike, Resistance 9–10, Revolutions per minute 100, Continuous Peddling. **Moderate Intensity 4 (2 min)** Modified dumbbell clean with widened base of support, using 2×10 lbs dumbbells ×1 min. Standing (knees backing to chair) bimanual overhead tricep press with 10 lbs dumbbell ×1min. **High Intensity 4 (1 min)** Sit to stand with 10 lbs dumbbell hold at chest ×1min (*cue, how many can you complete in 60 seconds). **Moderate Intensity 5 (2 min)** Seated diagonal cross body chops with 10 lbs dumbbell ×30 sec each side. Seated Up Downs (tap weight to floor, reach above head while sitting) with 10 lbs dumbbell ×1 min. **High Intensity 5 (1 min)** Modified burpees with chair for support ×1 min. **Cool Down (5 min):** Seated hip flexor stretch with overhead arm motion ×30 seconds each side. Spinal rotation stretch with deep breathing ×30 seconds each side. Seated hamstrings stretch ×30 seconds each side. Diaphragmatic breathing with Transverse abdominus and pelvic floor contraction ×10 repetitions. Pelvic Floor elevators ×5 repetitions.
Materials Needed for Aerobic Exercise	3 lbs dumbbell 5 lbs dumbbell 10 lbs dumbbell Upright exercise bike Green TheraBand^TM^ Wall for stability Chair	10 lbs dumbbell Upright exercise bike Chair
Balance Training	**Baseline Plan**	**Plan Progressed**
	**Order of exercises below was blocked (e.g., all exercises of Static Stance, then Dynamic Stance, Dynamic Gait, etc.)*. **Static Stance (in corner)** Feet together, eyes open on firm surface 2×30 sec. Semi-tandem 2×30 sec bilaterally. Neutral Stance eyes open on compliant surface 2×30 sec. Neutral stance eyes closed 2×30 sec (Practiced first with sunglasses as a precursor for dim-lit environment). Feet together eyes open on a compliant surface 2×30 sec. **Dynamic Stance (in corner)** Dynamic single limb stance with 2 finger hold, where P1 taps forward, side and back with toe (controlled), done bilaterally 3×10 repetitions. **Rest Break x 2 min** **Dynamic Gait Training (in hallway)** Warm up walking in hallway with normal walking cues for heel strike without assistive devices, but may touch wall with fingers bilaterally, ×2 laps. Walk with wide base of support ×2 laps. Diagonal stepping (stepping out at a 45 degree angle) ×2 laps. Cool down normal walking with cue for heel strike without assistive device but may touch walls with fingers bilaterally ×2 laps.	**Order of exercises below was variable (e.g., A few exercises of dynamic gait, followed by static stance, or a few dynamic stance, back to dynamic gait, etc.)*. **Static Stance (in corner)** Tandem Stance 2×30 sec bilaterally. 3/4 tandem stance with eyes open sunglasses donned, on firm surface 2×30 seconds bilaterally. Feet together stance, eyes open with vertical head nods, on compliant surface, 2× 30 seconds. **Dynamic Stance (in corner)** Dynamic single limb stance forward toe taps to 3-inch step, ×20 each leg. Dynamic single limb stance side toe taps to 3-inch step, ×20 each leg. Rocking forward and back while throwing dodge ball against wall and catching with right leg leading ×1 min. Rocking forward and back while throwing dodge ball against wall and catching with left leg leading ×1 min. Dynamic single limb stance where stance limb is on foam, and free limb engages in toe taps forward, side, back, and diagonal back ×10 each limb. **Vestibular (in corner)** Functional optokinetic motion with dodge ball. While in mini squat position, P1 looks at letters on dodge ball slowly moving ball horizontally vs. vertically each ×30 seconds (without symptoms). **Dynamic Gait (in hallway)** Warm up - Walking with heel strike 4×25 feet. Side stepping in mini squat position, alternating direction, no hand hold against wall, ×1min. Walking with sunglasses donned, no handhold against wall, ×1 min. Walking with head nods 2×25 feet. Walking with high-low touches to wall 4×25 feet. Retro ambulation with unilateral hand hold on wall 2×25 feet. Marching with turn slow clockwise, ×2, slow counterclockwise ×2. Marching with turn fast clockwise, ×2, fast counterclockwise ×2.
Materials Needed for Balance Training	Narrow Hallway Corner of room Compliant Surface (Towels) Sunglasses	Narrow Hallway Corner of room Compliant Surface (Towels) Sunglasses Dodge ball
	**Baseline**	**Post-Intervention**
Quantitative Results	Scale for Assessment and Rating of Ataxia at Home 10/24 Cerebellar Cognitive Affective Syndrome Raw 114/120 Patient Reported Outcome Measure in Ataxia 9/40 Timed Up and Go test 24.08 sec 30 second sit to stand test 10 repetitions Activities Specific Balance Confidence 65% Fatigue Severity Scale 8/49 Neurological Quality of Life Scale 38/40	Scale for Assessment and Rating of Ataxia at Home 9/24 Cerebellar Cognitive Affective Syndrome Raw 117/120 Patient Reported Outcome Measure in Ataxia 9/40 Timed Up and Go test 21.82 sec 30 second sit to stand test 11 repetitions Activities Specific Balance Confidence 69.4% Fatigue Severity Scale 8/49 Neurological Quality of Life Scale 40/40 Patient Global Impression of Change 7/7
Qualitative Excerpts	**Would you have joined this program if virtual sessions were not an option?** “No.” **What did you think about doing these sessions virtually?** *“It was great!”* **What was your motivation for wanting to join the program?** *“Helping to provide research data for ataxians, as well as for personal improvement in balance confidence and gait.”* **What were some tools/benefits you gained from this experience that you did not expect?** *“Breaking movements down into pieces and understanding more about the workings of the cerebellum.”* **Do you have any suggestions for us as to how we can improve this program?** *“It would be wonderful if all physical therapists who work with people with ataxia could include more education.”*	

**Case Example: Mid Stage Cerebellar Ataxia randomized to the Low Intensity prior to Balance Training Group**

The below case represents a brief case study on a participant enrolled in the PRIME-Ataxia pilot Randomized Controlled Trial who has mid-stage ataxia. This person was randomized to the low intensity exercise prior to balance training group for the intervention phase. Below, we outline the intervention from baseline through to their final session with progressive exercises. We also describe the materials this person used in their home setting, and how we adapted the intervention over telehealth specifically to their needs. For this intervention, we took into consideration the person’s disease stage, functional mobility and balance scores, level of fitness, as well as considerations to the environment in which they would be exercising (e.g., safety, materials, presence of a care-partner consistently, etc.). At the end of the table, we briefly discuss their individual outcomes, as well as their perspectives on engaging within this type of study.

**Table:**

**PRIME-Ataxia Pilot Randomized Controlled Trial: Mid-Stage Case 2**		
Group	Low Intensity exercise prior to Balance Training (LOW-BT)	
Case Detail	P2: Pt is a 62-year old White male, with genetic confirmation of Spinocerebellar Ataxia (SCA) type 6 diagnosed 5 years prior to this study. His symptoms began approximately 16 years prior, with imbalance during walking and while turning. He reports a family history of SCA type 6. P2 is an accountant, who resides with his wife (care-partner). He engages in light physical activity as tolerable with short walks in the community with his wife, otherwise he has no formal exercise routine currently. He has not had any form of rehabilitation since his diagnosis. For baseline mobility, P2 ambulates in the home and community with lofstrand crutches. P2 has had multiple falls in the past year and stated he often rolls his ankles when he falls. Additionally, his wife noted he tended to fall backward or lose balance if he bumps into objects. P2’s wife was on-site and actively participated in assessments and the intervention for purposes of safety and moral support for P2.	
Low-Intensity Exercise	**Baseline Plan**	**Plan Progressed**
	Karvonen Formula was used to predict target heart rate per session, and Borg Rating of Perceived Exertion Scale was used to target rating of perceived exertion per session. **Resting heart rate:** 70 bpm **Target Moderate heart rate reserve (<40%):** <110 bpm **Target Light rating of perceived exertion:** 1–3 **Warm up (5 min):** Seated diaphragmatic breathing ×5. Seated diaphragmatic breathing with concurrent arms up and out (abduction) ×5 repetitions. Seated diaphragmatic breathing with transverse abdominus contraction ×5 repetitions. Seated diaphragmatic breathing with transverse abdominus contraction followed by pelvic floor contraction ×5 repetitions. **Low-Intensity (20 min)** Seated diaphragmatic breathing with trunk rotation. On exhale P2 asked to rotate further. Hold 2×30 seconds bilateral. Seated side bending with arm overhead, 2×30 seconds hold bilateral. Seated chest stretch with foam roller 2×30 seconds. Seated trunk extension segmental mobilizations with foam roller ×12 repetitions with 2–3 second hold each. Standing Calf (gastrocnemius bias) stretch 2×30 seconds bilateral (holding onto wall). Standing Calf (soleus bias) 2×30 seconds bilateral. (holding onto wall). Seated (Foot) Arch Doming 2×10 repetitions bilateral. Seated Great toe extension and abduction 2×10 repetitions bilateral. Seated Great toe extension 2×10 repetitions bilateral. Seated other digit extension 2×10 repetitions bilateral. Seated alternating great toe vs other digit extension 2×10 repetitions bilateral. Seated ankle eversion 2×10 repetitions bilateral. Seated foot intrinsic muscle flexion (towel scrunches) 2×10 repetitions bilateral. **Cool Down (5 min):** Seated piriformis stretch 2×30 seconds bilateral. Seated hamstrings stretch 2×30 seconds bilateral. Seated hip flexor stretch with overhead reach 2×30 seconds bilateral. Seated adductors stretch 2×30 seconds bilateral.	Karvonen Formula was used to predict target heart rate per session, and Borg Rating of Perceived Exertion Scale was used to target rating of perceived exertion per session. **Resting heart rate:** 68 bpm **Target Light heart rate reserve (<40%):** <109 bpm **Target Light rating of perceived exertion:** 1–3 **Warm up (5 min):** Seated diaphragmatic breathing ×5. Seated diaphragmatic breathing with concurrent arms up and out (abduction) ×5 repetitions. Seated diaphragmatic breathing with transverse abdominus contraction followed by pelvic floor contraction with hold of subsequent second breath ×5 repetitions. **Low-Intensity (20 min)** Seated diaphragmatic breathing with trunk rotation. On exhale P2 asked to rotate further. Hold 2×30 seconds bilateral. Seated side bending with arm overhead, 2×30 seconds hold bilateral. Seated chest stretch with foam roller 2×30 seconds. Seated trunk extension segmental mobilizations with foam roller ×12 repetitions with 2–3 second hold each. Standing Calf (gastrocnemius bias) stretch 2×30 seconds bilateral (holding onto wall). Standing Calf (soleus bias) 2×30 seconds bilateral (holding onto wall). Standing (Foot) Arch Doming 3×10 repetitions bilateral. Standing great toe extension and abduction 2×10 repetitions bilateral. Standing Great toe extension 2×10 repetitions bilateral. Standing other digit extension 2×10 repetitions bilateral. Standing alternating great toe vs other digit extension 2×10 repetitions bilateral. Seated ankle eversion with green TheraBand^TM^ 2×10 repetitions bilateral. Seated Calf Raise with 5 lbs water jug 2×10 repetitions bilateral. Seated foot intrinsic muscle flexion (towel scrunches) 2×10 repetitions bilateral. **Cool Down (5 min):** Seated piriformis stretch 2×30 seconds bilateral. Seated hamstrings stretch 2×30 seconds bilateral. Seated hip flexor stretch with overhead reach 2×30 seconds bilateral. Seated adductors stretch 2×30 seconds bilateral.
Materials Needed for Aerobic Exercise	Towel Chair	Green TheraBandTM Towel Chair 5 lbs water jug
Balance Training	**Baseline Plan**	**Plan Progressed**
	**Order of exercises below was blocked (e.g., all exercises of Static Stance, then Dynamic Stance, etc.)* **Static Stance (in corner)** Feet shoulder width apart on carpet, eyes open 2×30 seconds. Feet shoulder width apart on carpet, eyes open with slow horizontal head turns 1×30 seconds. Feet shoulder width apart on carpet, eyes open with slow vertical head turns 1×30 seconds. Narrowed base of support (not touching) on carpet, eyes open with slow horizontal head turns 1×30 seconds. Narrowed base of support (not touching) on carpet, eyes open with slow vertical head turns 1×30 seconds. Feet shoulder width apart, eyes open, on foam mat 2×30 seconds. Narrowed base of support (feet not touching), eyes open, on foam mat with core activation 2×30 seconds. Feet together, eyes open, on firm surface, two fingers on wall 1×30 seconds. **Dynamic Stance (in corner)** Sit to stand on firm surface from raised seat height with cue for “nose over toes” due to retropulsion ×5 repetitions. Sit to stand with manual resistance to pelvis from care-partner ×5 repetitions. Sit to stand with cue for “nose over toes” ×5 repetitions. Standing with feet shoulder width apart, eyes open, on foam mat, with upper limb quick reaches forward and backward on cue 3×10 repetitions. Standing on one limb, sliding other limb forward, side, back 3×10 repetitions bilateral. **Rest Break: None**	**Order of exercises below was variable (e.g., A few exercises of dynamic gait, followed by static stance, or a few dynamic stance, back to dynamic gait, etc.)* **Static Stance (in corner)** Feet together stance, eyes open, on carpet, 2×30 seconds. Semi-tandem stance, eyes open, on carpet, 2×30 seconds bilateral. ¾ Tandem with small space between lower limbs, eyes open, on carpet, 2×30 seconds bilateral. **Dynamic Stance (in corner)** Medial to Lateral weight shifting 3×10 repetitions per side. Side stepping 1×10 repetitions 1 finger hold to wall (cue nose over toes). 2×10 repetitions side stepping without holding on (cue nose over toes). Anterior to posterior weight shifting (cue to keep pelvis level, 2×10 repetitions per side). Anterior to posterior stepping (rocking motion), 1 finger hold on wall, 1×10 repetitions bilateral. Anterior to posterior stepping (rocking motion), no support, 2×10 repetitions . Reactive balance in wide split stance – ball toss (central throws) in corner with care-partner 2×10 repetitions. **Vestibular** Functional optokinetic motion with tennis ball. While seated, P2 looked at letters on tennis ball while slowly moving ball in a throwing motion (mimic tossing ball with dog) 2×30 seconds without symptoms. Seated brock string exercises 3×10 repetitions. Seated Vestibular Ocular Reflex (VORx1) to medium size target on wall, against simple background 2×30 seconds, without symptoms. **Dynamic Gait (Near wall, with Care-Partner)** Walking with Lofstrand Crutches 2×30 feet. Walking with Lofstand Crutches wearing sunglasses (simulate dim-lit environment) 4×30 feet. Walking with Lofstrand Crutches cue for posture and to lead with nose over toes 4×30 feet. **Rest Break: None**
Materials Needed for Balance Training	Corner of room Compliant Surface (2cm thick yoga mat) Care-partner for manual resistance Chair (with pillow to add height)	Corner of room Wall Loftstrand Crutches Care-partner for ball toss Tennis ball Paper on wall with letter String with 3 beads (brock string) Chair
	**Baseline**	**Post-Intervention**
Quantitative Results	Scale for Assessment and Rating of Ataxia at Home 11.5/24 Cerebellar Cognitive Affective Syndrome Raw 102/120 Patient Reported Outcome Measure in Ataxia 27/40 Timed Up and Go test 24.08 sec 30 second sit to stand test 10 repetitions Activities Specific Balance Confidence 28.1% Fatigue Severity Scale 33/49 Neurological Quality of Life Scale 25/40	Scale for Assessment and Rating of Ataxia at Home 8.5/24 Cerebellar Cognitive Affective Syndrome Raw 113/120 Patient Reported Outcome Measure in Ataxia 26/40 Timed Up and Go test 21.82 sec 30 second sit to stand test11 repetitions Activities Specific Balance Confidence 31.3% Fatigue Severity Scale 33/49 Neurological Quality of Life Scale23/40 Patient Global Impression of Change 6/7
Qualitative Excerpts	**Would you have joined this program if virtual sessions were not an option?** *“Yes.”* **What did you think about doing these sessions virtually?** *“I was leery at first, but it worked out well!”* **What was your motivation for wanting to join the program?** *“I need to figure out this disease.”* **What were some tools/benefits you gained from this experience that you did not expect?** *“The knowledge that even though this disease progresses, I can still learn or re-learn how to get things back.”* **Do you have any suggestions for us as to how we can improve this program?** *“Just keep researching.”*	

## Appendix C

Supplementary Figure 1 Depiction of Repeated Measures Analysis of Variance for Secondary Measures of Digital Outcomes **Abbreviations:** 30 second Sit to Stand Test,30sec STS; high intensity exercise prior to balance training, HIGH-BT; low intensity exercise prior to balance training, LOW-BT; Timed Up and Go test, TUG. *Note*. Plot output from repeated measures analysis of variance whereby digitally recorded measures of the Timed Up and Go Test (S1A), 30 second Sit to Stand Test (S1B), Neutral Stance Postural Sway (S1C), Postural Sway of Feet Together Eyes Open Stance (S1D), Postural Sway of Feet Together Eyes Closed (S1E), and Postural Sway of Tandem Stance (S1F) are each plotted and show effects of group across three time points (Baseline Week 0, Baseline Week 8, Mid-Intervention Week 12, and Post-Intervention Week 16).
