# Supplementary figures and images for: Fatigue Limits Motor and Cognitive Improvements after High-intensity Exercise Prior to Balance Training over Telehealth in People with Spinocerebellar Ataxia
Source: Int J Telerehabil. 2025 Dec 12;17(2):6713. doi: 10.63144/ijt.2025.6713 (PMC12726854; doi:10.63144/ijt.2025.6713)

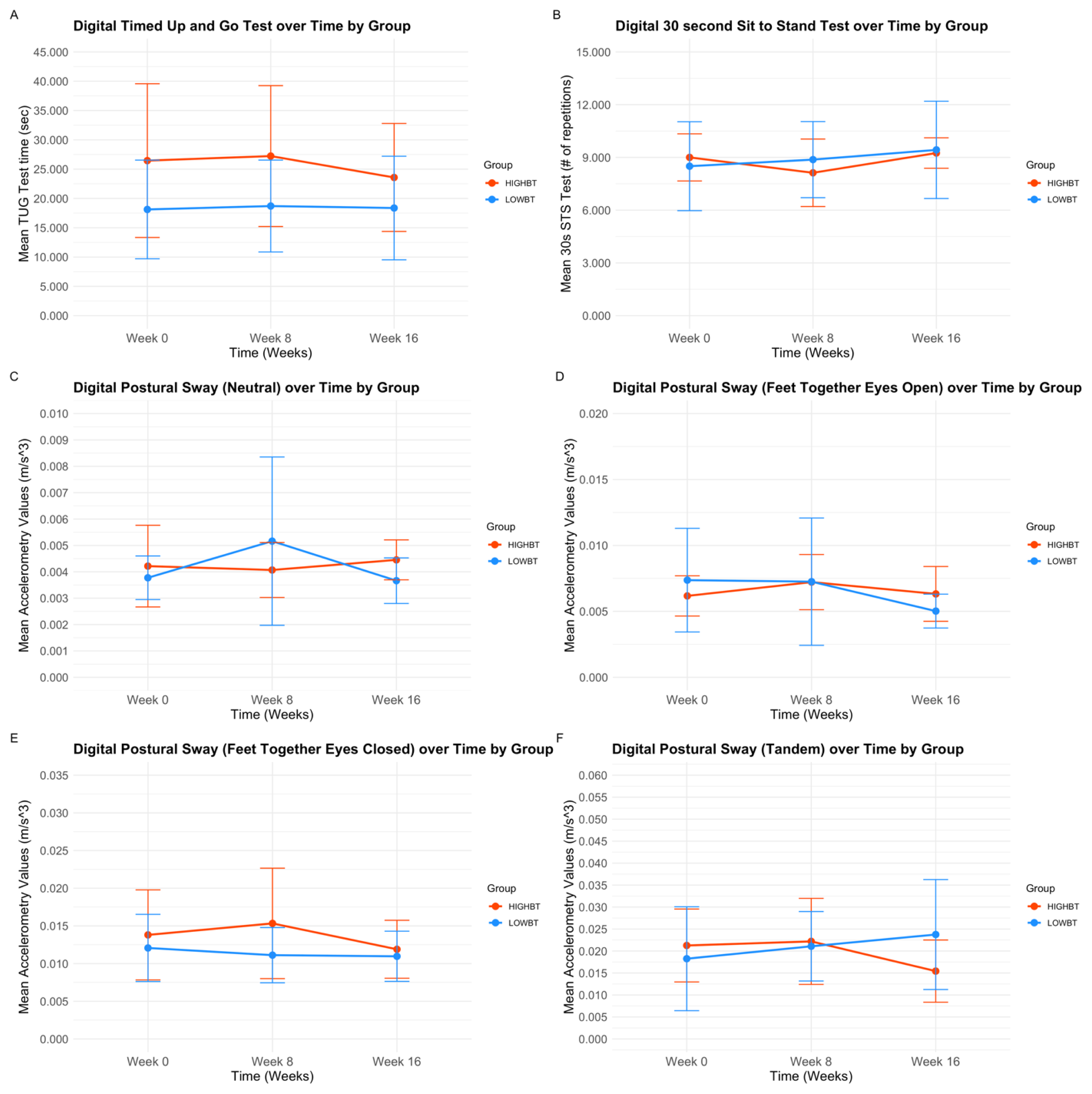

Supplement: Supplementary Figure 1 — Depiction of Repeated Measures Analysis of Variance for Secondary Measures of Digital Outcomes Abbreviations: 30 second Sit to Stand Test,30sec STS; high intensity exercise prior to balance training, HIGH-BT; low intensity exercise prior to balance training, LOW-BT; Timed Up and Go test, TUG. Note. Plot output from repeated measures analysis of variance whereby digitally recorded measures of the Timed Up and Go Test (S1A), 30 second Sit to Stand Test (S1B), Neutral Stance Postural Sway (S1C), Postural Sway of Feet Together Eyes Open Stance (S1D), Postural Sway of Feet Together Eyes Closed (S1E), and Postural Sway of Tandem Stance (S1F) are each plotted and show effects of group across three time points (Baseline Week 0, Baseline Week 8, Mid-Intervention Week 12, and Post-Intervention Week 16). [file ijt-17-2-6713s1.tif]
